# Ketoacidosis at onset of type 1 diabetes in children up to 14 years of age and the changes over a period of 18 years in Saxony, Eastern-Germany: A population based register study

**DOI:** 10.1371/journal.pone.0218807

**Published:** 2019-06-20

**Authors:** Ulf Manuwald, Olaf Schoffer, Janice Hegewald, Johann Große, Joachim Kugler, Thomas Michael Kapellen, Wieland Kiess, Ulrike Rothe

**Affiliations:** 1 Health Sciences/Public Health, Faculty of Medicine “Carl Gustav Carus”, TU Dresden, Dresden, Germany; 2 Center of Evidence-Based Healthcare, University Hospital “Carl Gustav Carus”, TU Dresden, Dresden, Germany; 3 Institute and Policlinic for Occupational and Social Medicine, Faculty of Medicine “Carl Gustav Carus”, TU Dresden, Dresden, Germany; 4 Institute of Sociology, Chemnitz University of Technology, Chemnitz, Germany; 5 Saxonian Network for Health Promotion "Sächsische Landesvereinigung für Gesundheitsförderung e.V.", Dresden, Germany; 6 Hospital for Children and Adolescents, Center for Pediatric Research, Department of Women and Child Health, University of Leipzig, Leipzig, Germany; McMaster University, CANADA

## Abstract

**Objective:**

The aim of this study was to examine the incidence trends of type 1 diabetes diagnosed with ketoacidosis in Saxony, Germany from 1999 to 2016.

**Methods:**

The population based Childhood Diabetes Registry of Saxony comprising valid data for all children aged 0–14 years diagnosed with type 1 diabetes from1999 to 2016 were used for the analyses. Direct age-standardized incidence rates were calculated and the effects of age, sex, calendar year, home districts and family history of any types of diabetes on the incidence were modelled using Poisson regression. Trend analyses for standard rate ratios of children with moderate and severe diabetic ketoacidosis versus children with type 1 diabetes with non-diabetic ketoacidosis were performed using join point regression.

**Results:**

The rate of ketoacidosis at the time of the type 1 diabetes diagnosis was high with 35.2% during the entire observation period in Saxony. The Poisson regression analysis indicated a statistically significant increased occurrence of diabetic ketoacidosis for younger age-groups, but no statistically significant differences between boys and girls. The join point trend analyses show that the proportion of severe and moderate ketoacidosis is increasing disproportionally to the increase in incidence of type 1 diabetes over the years.

**Conclusion:**

Due to the observed increasing incidence of diabetes as well of diabetic ketoacidosis, an educational prevention campaign is needed in Saxony as soon as possible to aid pediatricians, general physicians as well as general public to identify the early signs of type 1 diabetes.

## Introduction

Type 1 diabetes is an autoimmune disease often diagnosed during childhood. The characteristic classical symptoms of type 1 diabetes before the first diagnosis are polyuria, polydipsia, adynamia and weight loss. Type 1 diabetes results from the irreversible destruction of the ß-cells in the pancreas. Typically, most of the ß-cells in the pancreas (i.e. 60–80%) are destroyed and the insulin production disrupted before the initial type 1 diabetes diagnosis [1]. The etiology of type 1 diabetes is not yet known, but many factors, such as environmental factors [2, 3], such as ozone [4] and ultraviolet B irradiance and vitamin D levels in pregnant women [5], as well as genetic factors [6, 7], the intestinal microbiome [8], parental age, increasing birth weight [9] and increasing rates of cesarean sections are discussed as possible contributing factors. On the other hand, breast-feeding appears to reduce the risk of type 1 diabetes [10]. Due to the insulin deficiency, the glucose transport is interrupted. With the flow of glucose to the cells restricted, the body's energy can only be generated by breaking down fat which may be result in diabetic ketoacidosis.

The incidence rates of type 1 diabetes, as well as the rates of diabetic ketoacidosis at the time of the diabetes diagnosis vary internationally [11–13]. Große et al. 2018 analyzed 34 studies with diabetic ketoacidosis from five continents, and found the lowest rate of all newly diagnosed type 1 diabetes patients with diabetic ketoacidosis in Denmark (14.7%) and the highest rate in Saudi Arabia (79.8%) [14]. The Danish study enrolled a total of 129 children and adolescents under 17 years of age with newly diagnosed type 1 diabetes visiting one hospital from 2004 to 2006 [15]. The study from the Taif region of Saudi Arabia comprised 99 Saudi children under 13 years of age with newly diagnosed type 1 diabetes [16]. Countries with higher rates of incidence and prevalence of type 1diabetes have lower rates of diabetic ketoacidosis [14]. Some studies have shown decreasing rates of diabetic ketoacidosis in newly diagnosed children with type 1 diabetes [17–20], but other studies found increasing rates of diabetic ketoacidosis [21, 22]. In Saxony, type 1 diabetes incidence rates increased significantly between 1999 and 2014 (20.1 [95% CI 14.0; 26.1]) compared to the time before the reunification of Germany (7.9 [95% CI 6.8; 8.9]) [12]. The aim of this paper is to determine the rates of ketoacidosis at diabetes onset in Saxony are also changing over time.

## Materials and methods

### Data source

Patient data were available from the Childhood Diabetes Registry of Saxony [12], which provides valid data for all children aged 0–14 years diagnosed with type 1 diabetes since 1999. In Saxony, all children with diabetes are referred to a pediatric diabetologist working at one of 31 pediatric hospitals, and all of the pediatric hospitals regularly report new cases of diabetes to the registry. The registry data are checked each year for plausibility and the data of the hospitals are verified directly with the hospitals for completeness. The children’s date of birth, sex, birth weight, residential postcode, family history of (type 1) diabetes, date of manifestation, as well as clinical and biochemical data (e.g., pH and bicarbonate) at the time of the diabetes diagnosis were recorded using standardized forms.

Using the capture-mark-recapture method, the completeness of ascertainment of the Childhood Diabetes Registry of Saxony for children with diabetes under the age of 15 years has been estimated to be approximately 94% and 97%, respectively [11, 23]. Since the 1990s, type 1 diabetes is defined according to the EURODIAB criteria [24].

Ketoacidosis at the time of diagnosis was determined from the reported pH and bicarbonate values using the limit values for diabetic ketoacidosis from International Society for Pediatric and Adolescent Diabetes (ISPAD) [25]. We considered the severity of diabetic ketoacidosis by using the three categories defined by the ISPAD:

- Mild: venous pH<7.3 or bicarbonate<15mmol/l,
- Moderate: venous pH<7.2 or bicarbonate<10mmol/l
- Severe: venous pH<7.1 or bicarbonate<5mmol/l.

### Population at risk

Population data were obtained from the Statistical State Office of Saxony for the years 1999 to 2016. In Saxony, the population under the age of 15 years decreased from 578,355 (100%) in 1999 to 436,305 (75%) in 2005. Since 2005, the population under the age of 15 years has been slowly increasing to 527,543 in 2016.

### Statistical methods

Incidence rates were age-standardized according to the direct method [26] using the Standard New European Population (www.gbe-bund.de) for each calendar year of the observation period: 1999–2016. Age was grouped into three classes: 0–4, 5–9 and 10–14 years of age. Incidence data were presented as age-standardized incidence rates (SIR) per 100,000 person-years (PY) with 95% confidence intervals [CI] estimated using the normal approximation. Additionally, the annual incidence of ketoacidosis at diabetes onset was measured as the percentage of all children newly diagnosed with type 1 diabetes. These calculations were performed with the spreadsheet program Excel 2010.

The effects of age, sex, calendar year, home districts and family history of any types of diabetes were considered using Poisson regression models with the number of patients with type 1 diabetes as the offset. Also, a sensitivity analysis was conducted to determine if the self-reported duration (median≤2 weeks and median>2 weeks) of symptoms (polyuria, polydipsia, adynamia and weight loss) prior to the diagnosis was also predictive of ketoacidosis incidence. A dispersion test of inflated zeros was used to determine if the use of the Poisson model was justified. The zero-inflation test adduced no argument against the using of the Poisson distribution in the regression model. The Poisson regression analyses were performed using R 3.4.0.

Trend analyses for standard rate ratios of children with moderate and severe diabetic ketoacidosis versus children with type 1 diabetes without diabetic ketoacidosis were performed using join point regression, which is broadly used in cancer epidemiology. The join point regression was described by Boyle, P. and D.M. Parkin 1991 [27]. Annual percent change (APC) and the respective 95%CI were estimated for the complete observations period between 1999 and 2016. The fitted trend function was ln(y) = mx+b (x = year; b = intercept). Based on the slope parameter m, the annual percent change (APC) is the transformation (exp(m)-1)*100. Whether the trend changes over time was investigated for the complete time period. These calculations were performed with the Join Point Regression Program (Version 4.2.0.2, Statistical Research and Applications Branch, National Cancer Institute, Bethesda, Maryland, USA).

### Ethics statement

The Childhood Diabetes Registry of Saxony was approved by the Ethical Committee of the Medical Faculty of the University of Leipzig (Reg. Nr. 981) and written informed consent was obtained from all parents or guardians.

Only anonymous data was available for this analysis.

## Results

**Fig 1:**
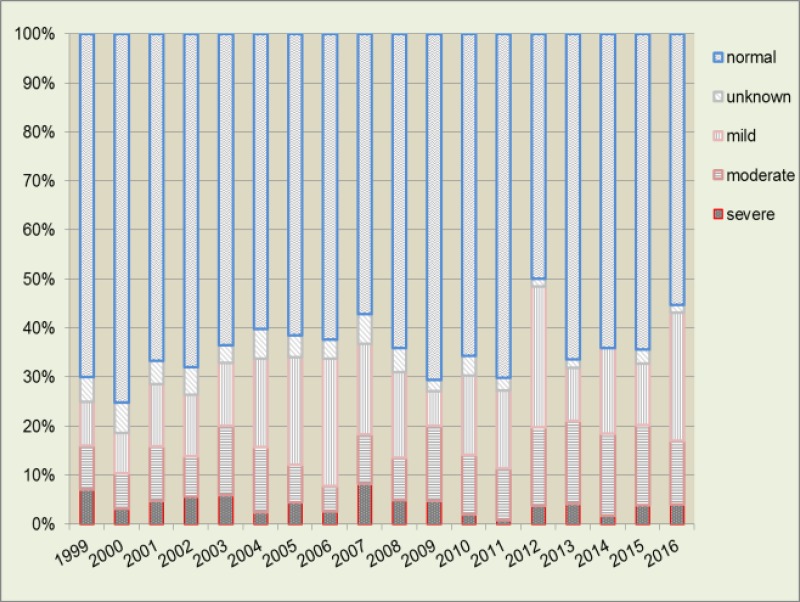
Diabetic ketoacidosis 1999–2016 [according to ISPAD].

In Saxony, there were a total of 1,759 incident cases of type 1 diabetes during the observation time period from 1999 to 2016. The majority of these cases (n = 1,122; 63.8%) had no ketoacidosis, while 292 (16.6%) had mild, 212 (12.1%) moderate, and 71 (4%) severe ketoacidosis at the time of diagnosis. For 62 (3.5%) of the children, there was insufficient information to determine the status of diabetic ketoacidosis. The mean symptom duration until the manifestation of type 1 diabetes was 15.5 days for the 0–4 years old children, 17.9 days for 5–9 years old children and 17.9 days for the 10–14 years old children. The yearly relative frequency of ketoacidosis at diabetes diagnosis and the rates of moderate and severe ketoacidosis per 100.000 PY are shown in Table 1 and Fig 1 and S1 Table. In Saxony, the rate of ketoacidosis at the time of a type 1 diabetes diagnosis was high with 35.2% during the entire observation period (1999–2016).

**Table 1:** Yearly cases of ketoacidosis at diabetes diagnosis and population at risk.

Year	Incident cases of type 1 diabetes	Ketoacidosis mild	Ketoacidosis moderate	Ketoacidosis severe	Moderate and severe ketoacidosis rates per 100,000 PY [95% CI]	Population at risk
1999	100	9	9	7	2.5 [1.2; 3.8]	578,355
2000	97	8	7	3	1.9 [0.7; 3.1]	550,835
2001	63	8	7	3	1.7 [0.6; 2.8]	522,225
2002	72	9	6	4	1.9 [0.7; 3.1]	494,070
2003	85	11	12	5	3.6 [1.9; 5.6]	470,594
2004	83	15	11	2	2.8 [1.3; 4.3]	451,952
2005	91	20	7	4	2.5 [1.0; 3.9]	436,305
2006	77	20	4	2	1.4 [0.3; 2.6]	437,421
2007	98	18	10	8	3.9 [2.1; 5.8]	444,508
2008	103	18	9	5	3.1 [1.5; 4.8]	454,198
2009	85	6	13	4	3.5 [1.8; 5.2]	464,584
2010	99	16	12	2	2.9 [1.4; 4.4]	476,168
2011	114	18	12	1	2.8 [1.3; 4.3]	480,220
2012	132	38	21	5	5.2 [3.2; 7.3]	488,364
2013	119	13	20	5	5.3 [3.2; 7.4]	496,028
2014	114	20	19	2	4.2 [2.4; 6.0]	504,802
2015	104	13	17	4	4.2 [2.4; 6.0]	518,224
2016	123	32	16	5	4.2 [2.4; 6.0]	527,543

### Regression and trend analysis

As shown in Table 2, the Poisson regression estimates indicated lower adjusted standardized rate ratios (SRR) of diabetic ketoacidosis for children in the 5–9 year old (SRR0.6; 95%CI 0.5–0.9) and 10–14 year old age-groups (SRR0.7; 95%CI 0.5–0.9) compared to the youngest children (0–4 years old). The children without any family history of diabetes were more likely to have been diagnosed with a ketoacidosis (SRR1.3; 95%CI 1.0–1.7).

**Table 2:** Results of the Poisson regression analysis in relation to ketoacidosis.

	Standardized rate ratio (SRR) [95% CI]
Year (continuous)	1.0 [0.9; 1.0]
Male	Reference
Female	1.2 [0.9; 1.5]
No family history of diabetes	1.3 [1.0; 1.7]
Age group 0–4 years	Reference
Age group 5–9 years	0.6 [0.5; 0.9]
Age group 10–14 years	0.7 [0.5; 0.9]
**Regional districts (where child resided)**	
“Bautzen”	Reference
“Chemnitz”	0.5 [0.3; 1.0]
“Dresden”	1.0 [0.6; 1.7]
Ore Mountains district “Erzgebirgskreis”	0.6 [0.4; 1.1]
Goerlitz “Görlitz”	0.7 [0.4; 1.4]
“Leipzig”	1.0 [0.6; 1.8]
Leipzig City “Leipzig Stadt”	0.8 [0.5; 1.4]
Meissen “Meißen”	0.9 [0.5; 1.5]
Middle Saxony “Mittelsachsen”	0.7 [0.4; 1.2]
North Saxony “Nordsachsen”	0.6 [0.3; 1.2]
Saxony Switzerland and Eastern Ore Mountains “Sächsische Schweiz-Osterzgebirge”	1.6 [1.0; 2.7]
Vogtland district “Vogtlandkreis”	0.9 [0.5; 1.6]
“Zwickau”	0.6 [0.3; 1.1]

With the exception of the borderline significant SRR estimate for the regional district Saxony Switzerland and Eastern Ore Mountains” (“Sächsische Schweiz-Osterzgebirge”; (SRR1.6; 95%CI 1.0–2.7)), no statistically significant differences between the various regions (reference: Bautzen) were observed. There was also no statistically significant difference in diabetic ketoacidosis rates between boys and girls.

The additional sensitivity analysis considering duration of the first symptoms until the manifestation of diabetes indicated that longer durations of symptoms prior to the diagnosis were not associated with an increased risk of ketoacidosis (SRR1.0; 95% CI 0.8–1.3).

The results of the trend analyses with age-standardized incidence rates using join point regression are shown in Table 3.

**Table 3:** Results of the trend analyses in relation to ketoacidosis using join point regression.

MODEL		Slope Estimate	Slope Std. Error	Annual percent changes (APC)	(95% CI)
Log-Linear Model of the age-standardized rates	Moderate+ severe ketoacidosis	0.05	0.0004	5.0	(2.6; 7.5)
	Without ketoacidosis	0.02	0.0041	1.9	(0.7; 3.2)
	All cases of type 1 diabetes	0.03	0.0002	2.8	(1.6; 4.1)
Log-Linear Model of the Standardized Rate Ratios	Moderate+ severe vs. without ketoacidosis	0.03	0.0135		

The join point log-linear model of age-standardized rates to determine if the incidence of type 1 diabetes and the manifestation of severe and moderate ketoacidosis at diagnosis were changing over time found higher APCs for severe and moderate ketoacidosis (APC5.0; 95%CI 2.6–7.5) compared to the overall incidence of type 1 diabetes and to incidence of type 1 diabetes without ketoacidosis (APC1.9, 95%CI 0.7–3.2). The log-linear model of the standardized rate ratios shows that the rate of diagnoses with moderate and severe ketoacidosis is increasing significantly faster the rate of diagnoses without ketoacidosis. As the permutation test found no join point for any of the models, there is no evidence to rule out a continuous trend.

## Discussion

Although the observed frequency of ketoacidosis at diagnosis observed in Saxony of 35.2% for the years 1999–2016 falls near the middle of the reported ranges reported by Große et al. (14.7%-79.8%) [14], we found that frequency of ketoacidosis at diagnosis increased disproportionately compared to the observed increases of type 1 diabetes incidence rates during the same period. This suggests that urgent actions are needed for to improve the timely detection of type 1 diabetes in Saxony. In Europe, although incidence rates for type 1 diabetes tend to be higher in the North (and West) and lower in the South (and East), rates of ketoacidosis are typically lower in the North and higher in the South [11, 13, 14]. This suggests that where type 1 diabetes is more prevalent, the sooner type 1 diabetes may be considered as a possible explanation of the manifesting metabolic symptoms. Große et al. [14] calculate in a meta-regression that a higher Human Development Index correlated with lower diabetic ketoacidosis rate. Although the incidence of type 1 diabetes in Saxony has increased three-fold since the reunification of Germany [12], it is still relatively low and classical symptoms probably recognized later. An earlier diabetes diagnosis might also be expected among families with a family history of diabetes, due to the family’s possible increased knowledge of diabetes symptoms. Although we observed an increased estimated SRR for families without any family history of diabetes, the results were statistically significant. Therefore, we can confirm that children with a family history of (type 1) diabetes have lower prevalence of diabetic ketoacidosis at initial diagnosis [28]. The Poisson regression resulted that younger children (0–4 years old) are significant more affected of ketoacidosis than older children. Similar results were found in a study of type 1 diabetes among Serbian children Vukovic et al [29].

For the most part, we observed no notable differences between the regional districts. The one difference observed between the regional district “Saxony Switzerland and Eastern Ore Mountains” and the other districts might be due to the lower concentration of medical care in the rural region. On the other hand, this result should be treated with caution and may be an accidental finding, since findings of a study in Poland confirmed not this observation [30].

Type 1 diabetes is often diagnosed late after symptoms of ketoacidosis are present. Therefore, the ketoacidosis rates at the time of diagnosis are high [31]. Timely diagnosis of type 1 diabetes is crucial for optimum care and avoid (early as well as late) complications [32]. The classical, but non-specific symptoms of diabetes (i.e. polyuria, nocturia, polydipsia, adynamia and weight loss) are often not identified as symptoms of a beginning diabetes by parents, teachers and educators of the child’s as well as by the first treating physicians/pediatricians.

In September 2016 the on-going newborn screening program–the Freder1k Pilot Study–started in Saxony [33] as a potential long-term method to improve early (pre-clinical) detection of type 1 diabetes in the future. Freder1k screens for the genetic risk of type 1 diabetes among newborn children in 21 of 31 clinics in Saxony. However, any improvement of the ketoacidosis rates cannot be detected at present, but only in the future. Further research is needed to determine the effect of the newborn screening program on the incidence rates of diabetes and ketoacidosis. Therefore, a populations based ketoacidosis preventions campaign is necessary as soon as possible in Saxony.

*Limitations of the study*: Although this study is based on the comprehensive registry data from 1999–2016, the type 1 diabetes remains a relatively rare disease. Therefore, the relatively small number of cases considered here might not have been enough to detect significant differences for some of the factors considered. Also, minimal data collected by the registry, so consideration of additional factors that might impact the timing of a diagnosis, such as the education of the parents, could not be considered. Another limitation is that we have no further information about the children, such as body mass index. Therefore, we could not consider further risk factors in the regression and the trend analyses. More variables may be better for the model fit and can improve the models’ ability to predict ketoacidosis rates.

## Conclusion

In conclusion, we observed troubling increases in the rates of ketoacidosis diagnoses in Saxony since 1999. Freder1k, the on-going newborn screening program in Saxony, has not yet had an effect on ketoacidosis at onset of diabetes.

In the future, the Freder1k study may reduce the rate of ketoacidosis among children who take part in the screening, but in the meantime a prevention program is needed.

A prevention program is urgently needed to improve the early detection of type 1 diabetes and to reduce the rate of ketoacidosis at diabetes onset. For example, an information campaign could increase the awareness of type 1 diabetes symptoms in the general public, and among child care workers and school teachers, as well as provide physicians with training to help them recognize the symptoms of type 1 diabetes early enough to prevent the potentially life-threatening metabolic disorder.

With the increasing incidence rates of diabetes in Saxony, the pediatric diabetologists who are chiefly responsible for the care of diabetes patients may also require additional support in order to continue to provide effective care.

## Supporting information

S1 Table Ketoacidosis stratified by sex, age group and family history. (DOCX)
