# Supplementary material for: Ketoacidosis at onset of type 1 diabetes in children up to 14 years of age and the changes over a period of 18 years in Saxony, Eastern-Germany: A population based register study
Source: PLoS One. 2019 Jun 20;14(6):e0218807. doi: 10.1371/journal.pone.0218807 (PMC6586407; doi:10.1371/journal.pone.0218807)
Supplement: S1 Table — (DOCX) [file pone.0218807.s001.docx]

**S1 table. Ketoacidosis stratified by sex, age group and family history.**

|  | Type 1 diabetes without ketoacidosis | Ketoacidosis mild | Ketoacidosis moderate | Ketoacidosis severe | Ketoacidosis unknown |
| --- | --- | --- | --- | --- | --- |
| **male** | 627 | 163 | 112 | 34 | 30 |
| **0-4 years old** | 133 | 37 | 34 | 8 | 10 |
| Diabetes in family history | 70 | 21 | 20 | 5 | 8 |
| Diabetes not in family history | 63 | 16 | 14 | 3 | 2 |
| **5-9 years old** | 219 | 36 | 26 | 11 | 7 |
| Diabetes in family history | 113 | 22 | 17 | 7 | 5 |
| Diabetes not in family history | 106 | 14 | 9 | 4 | 2 |
| **10-14 years old** | 275 | 90 | 52 | 15 | 13 |
| Diabetes in family history | 138 | 52 | 28 | 8 | 11 |
| Diabetes not in family history | 137 | 38 | 24 | 7 | 2 |
| **female** | 495 | 129 | 100 | 37 | 32 |
| **0-4 years old** | 70 | 27 | 26 | 11 | 6 |
| Diabetes in family history | 30 | 15 | 17 | 6 | 2 |
| Diabetes not in family history | 40 | 12 | 9 | 5 | 4 |
| **5-9 years old** | 199 | 45 | 39 | 11 | 13 |
| Diabetes in family history | 108 | 27 | 21 | 6 | 8 |
| Diabetes not in family history | 91 | 18 | 18 | 5 | 5 |
| **10-14 years old** | 226 | 57 | 35 | 15 | 13 |
| Diabetes in family history | 107 | 25 | 22 | 11 | 4 |
| Diabetes not in family history | 119 | 32 | 13 | 4 | 9 |
